# Fitness cost of a *mcr-1*-carrying IncHI2 plasmid

**DOI:** 10.1371/journal.pone.0209706

**Published:** 2018-12-26

**Authors:** Ke Ma, Yu Feng, Zhiyong Zong

**Affiliations:** 1 Center of Infectious Diseases, West China Hospital, Sichuan University, Chengdu, China; 2 Division of Infectious Diseases, State Key Laboratory of Biotherapy, Chengdu, China; 3 Center for Pathogen Research, West China Hospital, Sichuan University, Chengdu, China; University of Graz, AUSTRIA

## Abstract

IncHI2 is a common type of large *mcr-1*-carrying plasmids that have been found worldwide. Large plasmids could impose metabolic burden for host bacterial strains, we therefore examine the stability and fitness cost of a *mcr-1*-carrying 265.5-kb IncHI2 plasmid, pMCR1_1943, in *Escherichia coli* in nutrient-rich LB and nutrient-restricted M9 broth. Stability tests revealed that pMCR1_1943 was stably maintained with a stability frequency of 0.99±0.01 (mean ± standard deviation) after 880 generations in LB and 0.97±0.00 after 220 generations in M9 broth. Relative fitness (expressed as w, defined as relative fitness of the plasmid-carrying strain compared to the plasmid-free progenitor strain) was examined using the 24-h head to head competitions. pMCR1_1943 initially imposed costs (w, 0.88±0.03 in LB, 0.87±0.01 in M9) but such costs were largely reduced after 14-day cultures (w, 0.97±0.03 in LB, 0.95±0.03 in M9). The stable maintenance and the largely compensated cost after passage may contribute to the wide spread of *mcr-1*-carrying IncHI2 plasmids. To investigate potential mechanisms for the reduced fitness cost, we performed whole genome sequencing and single nucleotide polymorphism calling for the competitor strains. We identified that molecular chaperone-encoding *dnaK*, cell division protein-encoding *cpoB* and repeat protein-encoding *rhsC* were associated with the cost reduction for pMCR1_1943, which may represent new mechanisms for host bacterial strains to compensate fitness costs imposed by large plasmids and warrant further studies.

## Introduction

Colistin is the last resort of antimicrobial agents against most Gram-negative bacteria but colistin-resistant strains have emerged worldwide as a critical threat for clinical management and public health [1]. Colistin resistance can be mediated by the modifications of chromosomal genes or plasmid-borne mechanisms. *mcr-1* is the first reported plasmid-borne colistin resistance gene and mediates resistance to colistin by modifying the 4'-phosphoethanolamine of the lipid A on lipopolysaccharide [2]. *mcr-1*-carrying colistin-resistant strains have been identified in many countries, representing a global problem [3, 4]. *mcr-1* is commonly carried by plasmids of various replicon types, among which IncHI2 is relatively common [4, 5]. IncHI2 plasmids are large in size, typically > 200 kb. Such large plasmids may impose significant metabolic burden for host strains and may be lost during bacterial cell division of bacterial cells. We therefore performed a study to determine the stability and fitness costs of a large *mcr-1*-carrying IncHI2 plasmid.

## Material and methods

### The plasmid

A *mcr-1*-carrying IncHI2 plasmid, pMCR1_1943, was identified in an *Escherichia coli* strain from hospital sewage at West China Hospital in 2017. The complete sequence of pMCR1_1943 (265,538 bp) was obtained by using both the HiSeq X10 platform (Illumina, San Diego, CA, USA) and the long-read MinION Sequencer (Nanopore, Oxford, UK) and has been deposited into GenBank under accession no. CP027202. Of note, the IncHI2 plasmid also has an IncN replicon. The plasmid was transferred to azide-resistant *E*. *coli* strain J53 by conjugation at a 10^−6^ frequency per recipient cells as described previously [6]. The presence of *mcr-1* in transconjugants was confirmed by PCR with the primers CLR5-F (5ʹ-CGGTCAGTCCGTTTGTTC-3ʹ) and CLR5-R (5ʹ-CTTGGTCGGTCTGTA GGG-3ʹ) as described previously [2].

### Plasmid stability test

For testing the plasmid stability, *E*. *coli* J53 strain containing the plasmid was inoculated at 37°C overnight in 15 ml LB broth with 2 μg/ml colistin and 150 μg/ml sodium azide. These cultures were washed with saline solution (0.9% NaCl) several times to remove colistin and sodium azide. Aliquots (15 μl) were added to either 15 ml LB broth or 15 ml minimal media (M9 with 0.2% glucose, which is simply referred as M9 broth in this study) correspondingly. After incubation at 37°C overnight with shaking at 200 rpm, 100-μl samples were collected, diluted 1:10^4^ with LB or M9 broth correspondingly after being measured to the 0.5 McFarland standard, and were then streaked onto LB or M9 agar plates with and without 2 μg/ml colistin correspondingly, which constituted the initial (0-day) cultures. The initial inoculum density was about 1 × 10^2^ cfu/ml. Cultures were serially passaged for 14 consecutive days with a 10-μl fresh aliquot being transferred into 10 ml antimicrobial-free fresh LB or M9 broth (1:1000 dilution) every 8 h to keep the bacterial growth in the exponential phase. After 14-day cultures, 100-μl samples were collected, were diluted in LB or M9 broth after being measured to the 0.5 McFarland standard, and were streaked onto LB or M9 agar plates with and without 2 μg/ml colistin. The experiments were performed in triplicate. The stability frequency of plasmids was calculated by log_10_(*Ng*)*/*log_10_(*Nw*), where *Ng* and *Nw* represent densities of bacterial cells containing the plasmid and all bacterial cells in the media, respectively.

### Head to head competitions and fitness cost determination

The relative fitness (w) of strains carrying plasmids was defined as relative fitness of the plasmid-carrying strain compared to the plasmid-free progenitor strain and was determined in 24-h head to head competitions between plasmid-free J53 and the plasmid-containing transconjugant in both LB and M9 broth [7]. *E*. *coli* strains are usually carried by hosts for long time and therefore we also examined the impact of 14-day cultures on fitness costs by preforming two types of 24-h head to head competitions. One was between the initial (0-day) cultures of J53 with and without pMCR1_1943. The other was between 14-day cultures of J53 with and without pMCR1_1943, all of which were cultured independently for 14 days in the absence of antimicrobial agents in the plasmid stability test. For competition, the competitors were preconditioned in prewarmed LB or M9 broth for 24 h. After that, each strain cultures were measured to the 0.5 McFarland standard and a 10-μl aliquot of each competitor was mixed at a 1:1 ratio. The initial inoculum density of each competitor was about 2 × 10^3^ cfu/ml. The mixture was then inoculated in 10 ml of LB or M9 broth without colistin for 24 h at 37°C and 200 rpm. Experiments of head to head competition were performed in triplicate and were repeated three times [8]. Initial (*N*_*0*_) and final (*N*_*24*_) densities of each competitor were measured by selective (with 2 μg/ml colistin) and non-selective (without colistin) plating on LB or M9 agar plates. w was calculated using the equation, w = log_10_(*Ng*_*24*_/*Ng*_*0*_)/log_10_(*Nw*_*24*_/*Nw*_*0*_), where *Ng* and *Nw* are bacterial densities of plasmid-carrying and plasmid-free cells, respectively. A <1 w value suggests a fitness cost, while w >1 suggests a fitness advantage [9].

### Statistical analysis

Two-tailed *t* tests were used to compare the relative fitness cost between the initial 0-day and the 14-day cultures in the same culture media, which were performed using SPSS version 21.0 (IBM Analytics, Armonk, NY). All *P* values were two-tailed, and *P*<0.05 was considered statistically significant.

### Whole genome sequencing and single nucleotide polymorphism (SNP) calling

We sequenced the whole genome of J53 strains with pMCR1_1943 and compared the genome sequence of the strain cultured for 14 days to that of the same strain of the initial 0-day cultures to identify mutations of certain genes associated with the largely compensated fitness cost after 14-day cultures. To exclude the impact of 14-day culture in the broth, the original J53 strain without pMCR1_1943 was also subjected to 14-day cultures in LB and M9 broth and the cultures were also subjected to whole genome sequencing and SNP calling with 14-day cultures of J53 compared to the 0-day initial cultures of J53. For each 0-day initial and 14-day cultures of *E*. *coli* J53 with or without pMCR1_1943 in each of the two media (LB and M9), one bacterial colony on agar plates was selected randomly for genome sequencing. Genome DNA was prepared using the QIAamp DNA mini kit (Qiagen; Hilden, Germany) and was subjected to whole genome sequencing using the Illumina HiSeq X10 platform. Raw reads were trimming for adapter sequences and filtering for low quality reads (lower than Q20) using Trimmomatic v0.36 [10] and were then assembled using Unicycler v0.4.5 [11] in conservative mode with other settings as default. High-quality SNPs of 14-day cultured strains were identified by mapping their reads against the draft genome of their 0-day initial counterparts using snippy v4.0 (https://github.com/tseemann/snippy) with default settings. Function of genes with SNPs was assigned using the protein BLAST program (https://blast.ncbi.nlm.nih.gov/) against the reference protein database and was also predicted using Interpro (http://www.ebi.ac.uk/interpro).

For each 0-day initial and 14-day cultures of *E*. *coli* J53 with or without pMCR1_1943 in each of the two media, in addition to the colony that was subjected to whole genome sequencing, nine colonies were randomly selected from the three replicates (three colonies per replicate). The presence of SNPs in the colonies from each of the conditions (0-day or 14day cultures, with or without pMCR1_1943, and LB or M9 broth) were examined by PCR with self-designed primers (Table 1) and Sanger sequencing.

**Table 1:** Self-designed primers to verify the presence of SNPs.

Primer	Sequence (5'-3')	Target gene or region	Amplicon size, bp	Target strain
dnaK-L	CCAGACGTTTCGCCCCTATT	*dnaK*	2131	J53:pMCR1_1943 in LB
dnaK-R	CACGCTCTTCCGCTGTTTTG			
cpoB-L	CGAAGCGGCATACTCCAAAA	*cpoB*	894	J53:pMCR1_1943 in LB
cpoB-R	GCACGACACGACCAGAAATA			
fimD-L	AAATGACGGGCGTAATGGAA	Spacer upstream of *fimD*	135	J53:pMCR1_1943 in LB
fimD-R	AGCATTGTGTGTTTCGCTGG			J53 in LB
rhsC-L	GGTGGCGCAGATGCAAAGCC	*rhsC*	937	J53:pMCR1_1943 in M9
rhsC-R	ATTAACTCGCTCACATCACG			
gabD-L	GCCTTACACGCCGCATTTAA	*gabD*	1543	J53 in LB
gabD-R	CGGCGCTGCATTAACTCTTT			
mprA-L	GTTGTCTCTACCGCACCAGA	*mprA*	975	J53 in M9
mprA-R	TTTGCCGCTCTTCTTTACCG			
orf-L	CTCGCCTATGTGCTGGGTAC	orf	1977	J53 in M9
orf-R	GCTACAGAGGGTGATGGGGT			

Short reads of genome sequences of the strains in the present study have been deposited into Sequence Read Archive of GenBank under the accession no. SRR7031288, SRR7031297, SRR7031306, SRR7031313, SRR7031315, SRR7031316, SRR7031320, and SRR7031295.

## Results

### The *mcr-1*-carrying InHI2 plasmid was stable in *E*. *coli*

**Fig 1:**
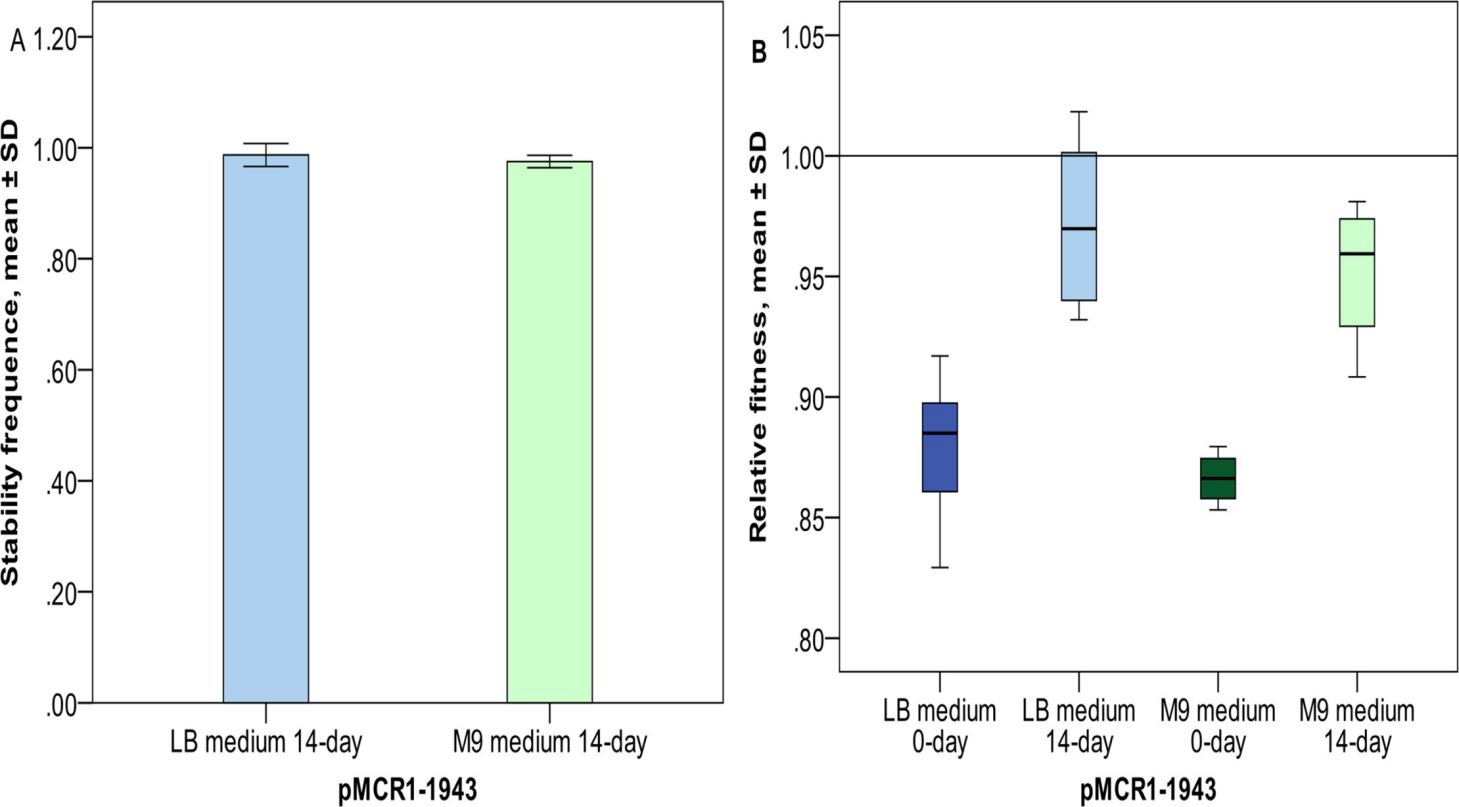
Stability and fitness costs of pMCR1_1943 in *E*. *coli* J53 strain. **1A,** stability, the experiments were performed in triplicate. The mean ± standard deviation (SD) of the stability frequency is shown. **1B,** relative fitness of J53 with pMCR1_1943 compared with J53 without any plasmids. 0-day refers to competitions between J53 with and without pMCR1_1943 at day 0, while 14-day refers to competitions between J53 with and without pMCR1_1943, both of which were independently cultured for 14 days. The experiments were performed in triplicate and each replicate was repeated in three times. The boxes represent relative fitness (w values; the mean ± SD) of the nine replicas.

Under the conditions described above, a 23-minute period of growth in LB broth and a 91-minute period of growth in M9 broth represents one generation, respectively. Cultures in 14 consecutive days corresponded to about 880 generations in LB broth and 220 generations in M9 broth, respectively. After 14-day cultures without any antimicrobial agents selecting for the plasmid, the stability frequency of the *mcr-1*-carrying IncHI2 plasmid was 0.99±0.01 (mean ± standard deviation) in LB broth and 0.97±0.00 in M9 broth (Fig 1A), respectively. This suggests that plasmid pMCR1_1943 was stably maintained in *E*. *coli* J53 strain in both nutrient-rich and -restricted media.

### The IncHI2 plasmid initially imposed fitness cost, which was largely reduced after 14-day cultures

Plasmid pMCR1_1943 indeed imposed fitness costs in both LB (w, 0.88±0.03; mean ± standard deviation) and M9 (w, 0.87±0.01) broth for the initial 0-day cultures of J53. However, after the strains (cultures of J53 with and without pMCR1_1943) were independently cultured for 14 days, such costs were largely reduced in LB (w, 0.97±0.03) and M9 broth (w, 0.95±0.03; Fig 1B). The difference of the w values between the 0-day and the 14-day cultures was significant in both LB (*t* = 6.482, *P*<0.001) and M9 broth (*t* = 8.925, *P*<0.001).

### Several single nucleotide polymorphisms (SNPs) were associated with the reduction of initial fitness cost imposed by the *mcr-1*-carrying IncHI2 plasmid

Whole genome sequencing generated 1.38 to 2.22 Gb clean reads for each strain, which were assembled into 115 to 152 contig (*N50*, 114,221 to 132,909 bp). Non-synonymous SNPs were found in *dnaK* (encoding a molecular chaperone) and *cpoB* (encoding a cell division protein) for strain J53 with pMCR1_1943 in LB broth and in *rhsC* (encoding an RHS repeat protein) for the same strain in M9 broth after 14-day cultures (Table 2). The three genes were all located on chromosome and no SNPs were identified in sequences belonging to the plasmid. These non-synonymous SNPs were also absent from J53 without pMCR1_1943. In addition, an SNP was identified in a spacer region (at 11 bp upstream of *fimD*, which encodes the outer membrane usher protein FimD) for strain J53 containing pMCR1_1943 cultured in LB media and a synonymous SNP was found in *rhsC* for the same strain culture in M9 broth (Table 2). The non-synonymous SNPs of *dnaK* and *cpoB* and synonymous SNPs of the spacer and *rhsC* were present in all of the nine colonies of J53 with pMCR1_1943 of the three replicates but were absent from all nine colonies of J53 without pMCR1_1943 as determined by PCR and Sanger sequencing. By contrast, the non-synonymous SNP of *rhsC* was present in five of the nine colonies of J53 with pMCR1_1943, while the remaining four colonies (from different replicates) did not have this SNP and any other additional SNPs in this gene. This non-synonymous SNP in *rhsC* was also absent from all nine colonies of J53 without pMCR1_1943.

**Table 2:** SNPs identified in the 14-day cultures of J53 strains containing pMCR1_1943 compared to the 0-day culture.

Strain	Media	SNP (aa sub)^1^	Gene or region	Product	NCBI Reference Sequence	Function^2^
J53:pMCR1_1943	LB	A→G (K55E)	*dnaK*	molecular chaperone DnaK	WP_108118574	
		C→A (-)	spacer^3^			
		T→A (T15A)	*cpoB*	cell division protein CpoB	WP_000097571	coordinates PBP1B and the Tol machines to maintain cell envelope integrity during division
	M9	G→A (S3N) A→G (-)	*rhsC*	RHS repeat protein	WP_096263337	Bacterial exotoxin, mediates intercellular competition
J53	LB	C→T (-)	spacer^3^			
		G→C (T208S)	*gabD*	NADP-dependent succinate- semialdehyde dehydrogenase I	WP_105285925	catalyzes the NADP+-dependent oxidation of succinate semialdehyde to succinate.
		C→T (-)	*mprA*	transcriptional regulator	WP_000378442	
	M9	C→T (A86*)	orf	DUF1116 domain-containing protein	WP_000083429	unknown

^1^aa sub, amino acid substitution; -, synonymous mutation

* introducing a stop codon.

^2^Function is only shown for proteins with non-synonymous mutations. Function assignation is from Interpro (http://www.ebi.ac.uk/interpro).

^3^The spacer region upstream of *fimD*, which encodes the outer membrane usher protein FimD. The C→A and C→T mutation occurs at 11 bp and 25 bp upstream of *fimD*, respectively.

For J53 without pMCR1_1943, non-synonymous SNPs in *gabD* (encoding an NADP-dependent succinate-semialdehyde dehydrogenase) and *mprA* (encoding a transcriptional regulator) and a SNP in a spacer region (at 25 bp upstream of *fimD*) were identified after 14-day cultures in LB broth, while an SNP resulting in a premature stop codon in an open reading frame (orf; encoding an DUF1116 domain-containing protein) was found after cultures in M9 media. Nonetheless, none of these SNPs were present in colonies of J53 with pMCR1_1943.

## Discussion

pMCR1_1943 is a large IncHI2 plasmid carrying *mcr-1* but could stably maintained in *E*. *coli* J53 strain in both nutrient-rich and -restricted media. Examining its complete sequence, we found that pMCR1_1943 had genes encoding plasmid partitioning (*parA-parB*) and a toxin-antitoxin postsegregation killing system (*hipA-hipB*), both of which can contribute to plasmid stability [12–14]. This may provide an explanation for the stable maintenance of the plasmid in *E*. *coli*.

Plasmid pMCR1_1943 initially imposes fitness cost for the host strain. It has been widely described the carriage of plasmid could impose fitness cost but the mechanisms of the cost remain largely unclear [15]. Nonetheless, a few potential mechanisms have been proposed including the metabolic burden imposed by plasmid replication, the consumption of resources for the expression of plasmid-encoded genes, synthesis of the plasmid conjugation apparatus, alteration in the expression of host genes, disruption of host genes or fine-tuning cellular pathways, and other metabolic effects such as the introduction of efflux pumps able to pump out important biomolecules [16–19]. Due to it large size (265,538 bp), it may be not surprising that plasmid pMCR1_1943 initially imposes fitness cost for the host strain. However, it is interesting that after 14-day passage, the fitness cost was largely reduced. Previous studies have also demonstrated that although plasmids initially impose fitness cost to host strains, the cost could be compensated or is even reversed to fitness advantage after passage of hundreds of generations [8, 20–23]. The compensation of fitness could be due to mutations in the bacterial host chromosome [20, 24], in plasmids [8, 25] or in both [21, 22]. The genes with mutations or the exact location of the mutations in the chromosome and plasmids were not indicated in most of these studies [8, 20–22]. However, mutations in plasmid replicon [25] and chromosomally-located global regulator genes [24] have been found to alleviate fitness cost imposed by plasmids. In addition to mutations, deletions of genetic components on plasmids due to insertion sequences [26] and acquisition of genes that are able to contribute to plasmid stability by plasmids [27] has also been identified as mechanisms to compensate fitness cost due to plasmids.

The mechanism responsible for the reduction of fitness cost imposed by pMCR1_1943 remains unclear. To untangle the potential mechanisms, we then performed comparative genomics and found SNPs in several genes. Interestingly, the genes associated with reduced fitness cost are different between cultures in LB and M9 broth, suggesting that the same host strain may employ different strategies to adapt different environment for reducing the burden imposed by the carriage of a large plasmid. Among the genes identified, the product of *dnaK* has been reported to involve in chromosomal DNA replication through the interaction with the DnaA protein and other yet-to-be-identified mechanisms [28]. *cpoB* encodes a cell division coordinator to maintain the cell envelope integrity during division [29]. Nonetheless, the association of the two genes with the reduction of fitness cost imposed by a plasmid has not been document before and may represent new mechanisms. Although the association is not equal to causality, the new associations found here warrant further investigations. The function of *rhsC* has not been fully understood but may involve in mediating exports [30]. Of note, the non-synonymous SNP in *rhsC* was absent in four of the nine colonies of strain J53 containing pMCR1_1943 cultured in M9 media, suggesting that this SNP is not necessarily associated with the reduction of fitness cost under nutrient-restricted conditions.

This study has notable limitations. First, we only performed whole genome sequencing for one bacterial colony per environment (nutrient-rich or -restricted). Without sequencing other biological replicas, we were unable to investigate parallel evolution among biological replicas to identify mutations under positive selection as demonstrated previously [31, 32]. Second, we only looked the interaction of a single plasmid with a single host strain. Different strains and species have different evolvability, which may have significant impact on plasmid adaptation [26]. Third, we did not perform RNA-seq to untangle mechanisms involving gene expression as shown before [33].

In conclusion, the *mcr-1*-carrying IncHI2 plasmid is stably maintained in *E*. *coli* although it is large in size. The *mcr-1*-carrying IncHI2 plasmid imposed fitness costs initially but the cost was largely compensated after long-term culture. The stable maintenance and the largely compensated cost after passage may contribute to the wide spread of *mcr-1*-carrying IncHI2 plasmids. Several genes were newly identified to be associated with the reduction of the cost imposed by the IncHI2 plasmid.
